# Sensory Consumer and Descriptive Analysis of Steaks from Beef Animals Selected from Tough and Tender Animal Genotypes: Genetic Meat Quality Traits Can Be Detected by Consumers

**DOI:** 10.3390/foods10081911

**Published:** 2021-08-17

**Authors:** Maurice G. O’Sullivan, Ciara M. O’Neill, Stephen Conroy, Michelle J. Judge, Emily C. Crofton, Donagh P. Berry

**Affiliations:** 1 Sensory Group, School of Food and Nutritional Sciences, University College Cork, T12 YN60 Cork, Ireland; ciara.oneill@mycit.ie; 2 Irish Cattle Breeding Federation, Highfield House, Shinagh, Bandon, P72 X050 Co. Cork, Ireland; Sconroy@icbf.com (S.C.); Michelle.judge@teagasc.ie (M.J.J.); 3 Teagasc, Ashtown Food Reseach Centre, Ashtown, D15 DY05 Dublin, Ireland; Emily.Crofton@teagasc.ie; 4 Teagasc, Animal & Grassland Research and Innovation Centre, Moorepark, Fermoy, P61 P302 Co. Cork, Ireland

**Keywords:** consumer, descriptive, sensory, tough, tender, beef, steak, genotype, prediction

## Abstract

The objective of the present study was to determine if animals who were genetically divergent in the predicted tenderness of their meat actually produced more tender meat, as well as what the implications were for other organoleptic properties of the meat. The parental average genetic merit for meat tenderness was used to locate 20 “Tough genotype” heifers and 17 “Tender genotype” heifers; M. longissimus thoracis steaks from all heifers were subjected to sensory affective analysis (140 consumers) and sensory profiling using two trained sensory panels. All sample steaks were treated identically regarding pre- and post-mortem handling, storage, cooking and presentation (i.e., randomised, blind coded). For the affective consumer study, eight steaks were sectioned from the same location of the striploin muscles from each of the heifers. In total, 108 steaks from the Tender genotype and 118 from the Tough genotype were tested in the consumer study to determine the preference or liking of these steaks for appearance, aroma, flavour, tenderness, juiciness and overall acceptability. The consumer study found that the Tender genotype scored higher (*p* < 0.0001) for liking of tenderness, juiciness, flavour and overall acceptability compared to the Tough genotype. Similar results were generally found for the separate consumer age cohorts (18–64 years) with lower sensory acuity in the 65+ age cohort. For the descriptive analysis, the Tender genotype scored numerically more tender, juicy and flavoursome, although the differences were only significant for one of the panels. The critical outcome from this study is that parental average genetic merit can be used to pre-select groups of animals for tenderness, which, in turn, can be detected by consumers.

## 1. Introduction

A consumer’s decision to purchase beef is guided by the perception of healthiness along with a variety of sensory characteristics including colour, appearance, tenderness, juiciness, aroma and flavour [1]. Of course, colour and visible fat are also important quality cues at the point of sale, since an appealing appearance raises consumer interest. However, when consuming at home after cooking, consumers experience the sensory qualities of tenderness, flavour and juiciness, and only then conclude whether the food will be re-purchased. Additionally, the major background cues for assessors are safety, nutrition, sustainability and ethics [2,3,4]. Thus, meat quality characteristics play a major role in consumers’ meat purchasing decisions, with the most fundamental considered being expected tenderness, juiciness, flavour, appearance and safety [5]. Tenderness has often been described as the most important factor in terms of the high eating quality of meats, especially beef [6,7]. It has been shown that a certain level of tenderness is crucial in order for the meat to be deemed acceptable [8]; tenderness is such an important quality attribute to consumers that they are willing to pay more for greater tenderness [9,10].

Genetic differences in meat quality exist represented both by inter-breed and intra-breed differences [11]. From a review of 10 available studies on meat tenderness in cattle, Berry et al. (2017) [12] documented a median heritability estimate of 0.23 for tenderness assessed by trained panels; this therefore suggests that after accounting for differences in systematic environmental effects (e.g., contemporary group, gender, age), 23% of the remaining within-breed variability in tenderness was due to additive genetic differences. Therefore, an efficient and effective breeding program can improve the mean meat tenderness of a population; similarly, the moderate heritability suggests that animals divergent in genetic merit for tenderness should display phenotypic differences. Such a phenomenon, if proven successful, could be extremely useful for meat processors and consumers. However, no study exists globally that has phenotypically characterised the sensory properties of meat samples divergent in genetic merit for sensory tenderness. The current study aimed to fill this void by characterising the meat from crossbred heifers who were predicted, based solely on ancestry, to produce either tough or tender steaks. The resulting steaks (M. longissimus dorsi) were treated identically regarding pre- and post-mortem handling, storage, cooking and presentation to 140 consumers and two trained descriptive panels. Characterisation metrics of interest included appearance, aroma, flavour, tenderness, juiciness, and overall acceptability. Thus, the presented study explored whether sensory assessors, trained and untrained consumers of beef, could detect differences (blind) in tough and tender samples, which were pre-determined from animals selected genetically with these meat quality traits. 

## 2. Methods and Materials

### 2.1. Source of Beef and Pre-Mortem Management

A genetic evaluation was undertaken for meat tenderness in Mix99 [13] based on the available dataset, statistical model and variance components described in detail by Berry et al. (2021) [12]. In brief, sensory data from trained panellists were available on 5380 animals consisting of 1579 young bulls, 1966 steers and 1835 heifers; the animals originated from 748 sires with the mean number of progeny per sire being 6.9. Data from two sensory locations, each with their own sensory panels, contributed to the database. The linear model fitted in the genetic evaluation was determined as per Berry et al. (2021) [12] and Judge et al. (2021) [11]:

Y = HSD + DL + GENDER + a + e(1)

where Y is the dependent variable of mean tenderness across all trained panellists; HSD is the fixed effect of the herd by date of slaughter; DL is the fixed effect of the date by location of sensory analysis; and GENDER is the gender of the animal (i.e., bull, steer or heifer); a is the additive genetic effect ($a\;\sim \;N(Qg,A\sigma_{a}^{2}$), where Q is a matrix relating a to genetic groups, g is a vector of genetic group means, **A** is the numerator relationship matrix and $\sigma_{a}^{2}$ is the additive genetic variance; and e represents the residual term, where N(0,**I**$\sigma_{e}^{2}$) with $\sigma_{e}^{2}$ represents the residual variance and **I** is an identity matrix. The heritability of mean tenderness score was assumed to be 0.16 as estimated by Berry et al. (2021) [12] using this dataset. A pedigree file consisting of all phenotyped animals and their ancestors was used; founder animals were allocated to genetic groups. Therefore, estimated breeding values (EBVs; estimates of genetic merit) for all sires (as well as other animals) were available; sires, divergent in genetic merit for tenderness based on data from at least five phenotyped progeny, were identified as potential sires with the validation of heifers to be used in the present study.

The Irish Cattle Breeding Federation (ICBF) operate the Irish national beef breeding program on behalf of the Irish industry. Semen of young test bulls is distributed to participating beef producers as part of the Gene Ireland breeding program; hence, a strong relationship exists between these herds and the ICBF, with the latter routinely purchasing cattle for performance tests from these producers. Therefore, the identified sires divergent in genetic merits for tenderness with progeny in these herds were considered. Divergence in sire EBV was sought while remaining cognisant of minimising the over-representation of any one breed in either a high or a low genetic merit stratum; this is because breed differences exist in meat tenderness [11]. A total of 40 heifers from 14 different sires were identified from as few herds as possible; all animals were purchased and taken to the ICBF performance test station at, on average (standard deviation), 435 (SE = 27) days of age. Following the genotyping of all animals, a total of 37 animals with verified sire identity were available; these were stratified into genetically Tender (*n* = 17) versus genetically Tough (*n* = 20) genotypes based on their sire’s EBV. All heifers were fed a total mixed ration ad libitium comprised of a concentrate, hay and water fresh-weight ratio of 10:3:9, as described in detail by Kelly et al. [14], and were slaughtered at a mean (SD) of 531 (27) days of age.

### 2.2. Meat Sampling

Animals were slaughtered and after 48 h post-mortem hanging, 8 steaks (2.54 cm thick) were cut from a deboned section of the longissimus thoracis (striploin) muscle on the right-hand side of the carcass. All samples were aged for 21 days at 4 °C before being transferred to a commercial freezer, individually vacuum-packed and held at −20 °C.

### 2.3. Consumer Analysis

Consumer Demographics: Beef consumers (*n* = 140) of varying age groups (18–65+) and genders (58% F, 42% M) were recruited via email and participated in this consumer study, which took place in the School of Food and Nutritional Sciences in University College Cork, Cork, Ireland. Consumers were asked to provide demographic information prior to tasting including information on age group, nationality, gender, occupation and steak cooking preference.

Sensory Evaluation: An unstructured 10 cm scale was used for consumer analysis and the tested attributes were as follows: liking of aroma (0 = dislike extremely, 10 = like extremely), tenderness (0 = not tender, 10 = very tender), juiciness (0 = not juicy, 10 = very juicy), liking of flavour (0 = dislike extremely, 10 = like extremely) and overall liking (0 = dislike extremely, 10 = like extremely). In total, 14 sensory sessions were carried out with 10 consumers per session and 4 samples per consumer per treatment.

Sample Preparation and Presentation: Striploin steaks were thawed by placing the frozen steaks in a chill (4 °C) for 24 h prior to cooking. Steaks were then cooked on an open clam shell grill (Velox CG-6 400 V 3 Phase Model) using a teaspoon of sunflower oil at 210 °C. Steaks were cooked for 1 min on each side to seal the meat and then turned every 2 min until cooked. The internal temperature was continually probed until 71 °C was reached. The steaks were then labelled, wrapped in aluminium foil and allowed to rest for 3 min. Steaks were cut into portions of 1.27 cm × 1.27 cm × 2.54 cm, ensuring that no gristle was present, the portions and two randomised portions from each sample were wrapped in aluminium foil, labelled with the corresponding 3 digit code and served to panellists.

Experimental Design: Each session contained a mix of Tough and Tender genotypes that were served blindly in a randomised order. Panellists received a dummy sample at the beginning of each session to reduce first order biases, assist in panellist standardisation and enhance panellist calibration before each session.

MQ4 Score: In addition to the scores provided by the consumers, a weighted eating quality score, MQ4 score, was calculated for each sample using the Australian MSA model (0.3 TE + 0.1 JU + 0.3 FL + 0.3 OL). Thus, a combined satisfaction score (MQ4 score) was also calculated by combining four sensory variables assessed by consumers, including tenderness (TE), juiciness (JU), flavour liking (FL) and overall liking (OL) [15]. In this system, beef muscles are categorised into one of four grades according to their MQ4 score: unsatisfactory (ungraded), satisfactory everyday quality (3*), better than everyday quality (4*) or premium quality (5*) [15].

### 2.4. Trained Sensory Panels

Sensory analyses of steak samples were undertaken by a commercial company, at Sensory Location 1, and by a sensory laboratory at Sensory Location 2, using trained sensory panels (7–8 individuals). The training of panellists for profiling meat description texture attributes was according to the American Meat Science Association (2015) [16]. The protocol for sensory analysis differed slightly between the two locations and is outlined in detail by Judge et al. (2021) [11].

Sample Preparation and Presentation Location 1: Frozen samples were thawed in a chill (4 °C) 24 h prior to analysis. Steaks were tempered at ambient without packaging 60 min prior to being fried on a “Mirror Griddle Model M1000E ENG” to an internal temperature of 71 °C. Steaks were rested and then cut into 1.27 cm × 1.27 cm × 2.54 cm portions (no gristle) and presented to each panellist while hot.

Sensory Evaluation: Each panellist was directed to cut the steak piece in half across the cooked surface and evaluate the sample in the same fashion (duplicate) using the assessment sheet provided. The steak sample was scored for tenderness, juiciness and flavour using a structured scale [11].

Sample Preparation and Presentation Location 2: Sensory samples were prepared identically as for location one. However, steaks were fried on a CG6 Velox grill to an internal temperature of 71 °C, wrapped in aluminium foil and rested for 2 min before cutting, similarly to Location 1.

Sensory Evaluation: Two randomised portions from each steak were wrapped in aluminium foil, labelled with the corresponding three-digit code and served to the panellists. A warm-up sample was served at the beginning of every session to reduce first-order bias and assist with the calibration of the panellists. Panellists were asked to score the samples using the assessment protocol defined during panel training, and to expectorate the sample into a cup/bowl [11]. The first steak sample portion was scored for tenderness, juiciness, beef flavour and chewiness. The second piece of the same sample was assessed in the same way to confirm the scores given to the first piece. Standard sensory practices, such as blind tasting and the randomisation of samples, were used during the assessment [11].

### 2.5. Statistics

The association between the genetic merit stratum (i.e., Tender versus Tough) and each of the mean tenderness, juiciness, flavour and chewiness (only for Sensory Location 2) values was determined using the linear mixed model PROC MIXED of SAS (SAS Institute Inc., Cary, NC. USA) for each sensory location separately. The sire was included as a random effect and the genetic merit stratum was included as a fixed effect; neither age nor heterosis coefficients were associated with the sensory-dependent variables and, because all animals were slaughtered on the same day, and underwent sensory analysis together on the same day, there was no need to adjust for either effect as per previous models [12,17]. The least squares means of the different sensory characteristics for the Tender and Tough genotypes were estimated from the model and compared. For the consumer analysis, the difference between the Tough and Tender genotypes across all data, as well as within each age cohort, was determined using a two-tailed distribution *t*-test.

## 3. Results and Discussion

The mean (standard deviation) EBV of the sires in the Tender and Tough genotypes was 0.21 (0.06) and −0.10 (0.11), respectively; this represents an average difference of 1.86 within-breed genetic standard deviation units [12]. Using the within-breed genetic standard deviation for the population, this equates to comparing, within breed, the top 42% of animals versus the bottom 42% of animals. The reason for such a relatively small selection intensity was the requirement for representation of common breeds in both genotypes so as to avoid a simple breed comparison; significant breed differences in tenderness do exist [11]. The Tender genotype heifers were sired by five different bulls from the Angus, Limousin, Hereford and Belgian Blue (×2) breeds; the Tough genotype heifers were sired by eight different bulls from the Angus, Limousin (×2), Hereford, Belgian Blue, Charolais and Simmental (×2) breeds. The mean reliability of the EBVs for the Tender and Tough genotypes was 0.42 and 0.50, respectively.

**Figure 1:**
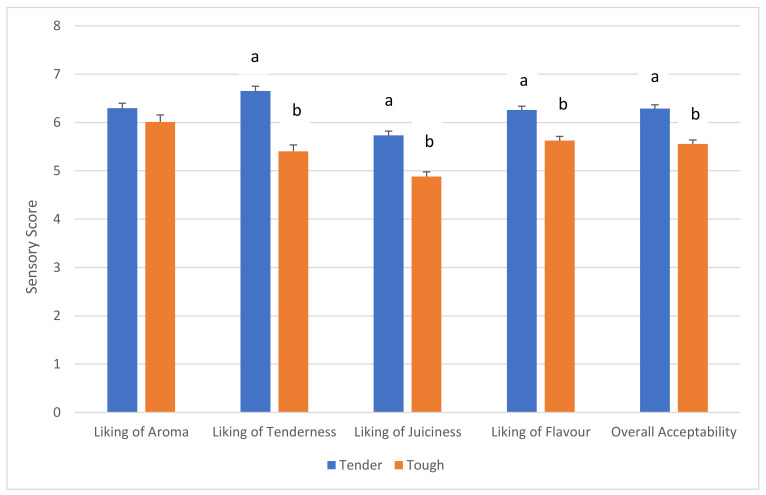
Consumer evaluation (hedonics) of beef from the Tough and Tender genotypes. Mean data with standard error of the mean (SEM). Lower case letters (a,b) denote significant differences (*p* < 0.0001) between columns.

The cumulative data from the consumer study revealed that the Tender genotype scored (*p* < 0.0001) higher for liking of tenderness, juiciness, flavour and overall acceptability compared to the Tough genotype (Figure 1). Similar results were detected for all age groups, although a significant difference in the aroma of Tough versus Tender genotypes was not always detected (Table 1). However, all age groups scored the Tender genotype samples higher for liking of Tenderness. Additionally, the Tender genotype samples, as well as scoring higher for liking of tenderness, also scored higher for juiciness, flavour and overall acceptance; this is not unexpected given the moderate to strong phenotypic and genetic correlations that exist between tenderness, juiciness and flavour in cattle striploins [13]. Chong et al. (2019) [18] observed that tender samples of beef also scored higher for liking of juiciness, liking of flavour and overall acceptance.

In more recent years it has become increasingly apparent that consideration should also be given to meat flavour, which has been found to be the most important factor affecting consumers’ meat buying habits and preferences when tenderness was held constant [19,20,21]. Santos et al., [22] states that ‘if the flavour is not acceptable, beef is rejected regardless of the other attributes’ and, similarly, ‘if tenderness is not acceptable, beef is also rejected regardless the other attributes’.

The juiciness of meat is also of great importance as a quality factor in the overall eating experience of muscle foods. Juiciness is the feeling of moisture in the mouth during chewing. It is a combination of moisture chewed out of the meat and saliva production mixed into the meat. The juiciness of meat depends on both the raw meat quality, but also the cooking procedure employed. Later in the chewing process, juiciness becomes a combination of moisture from the meat and saliva production [23]. Juiciness is highly correlated with tenderness, and when tenderness is within the consumer liking range, the flavour is the most important attribute contributing to the overall liking of beef [24]. However, juiciness liking can be easily compensated or influenced by flavour and tenderness (medium cuts of steaks), meaning that juiciness is an attribute that contributes less to the overall liking [25].

The age cohorts 18–24, 25–34, 45–54 and 55–64 scored liking of juiciness, liking of flavour and overall acceptability higher for the Tender genotype samples compared to the Tough genotype samples. All other age cohorts also scored these attributes numerically in a similar fashion, albeit with no significant inter-genotype differences. It is not clear why the 35–44 age cohort did not significantly perceive differences between the treatments, although attribute scores were higher for the Tender genotype treatment. However, the results for the 65+ cohort are in partial agreement with those found by Conroy et al. (2017) [23], who investigated the difference in textural preference in various age cohorts for beef steak tenderness. They found that consumers aged between 18 and 70 years successfully detected the attribute differences between the meat sample groups of moderately Tough (MTH) and Tender (TR) steak samples. However, attribute blindness, particularly for texture, was observed in sensory subjects older than 71 years. These authors [23] postulated that this was due to a loss of textural acuity for meats in this cohort perhaps due to poor dentition [26], sensory decline as the aging process occurs [27], social factors and medications, as well as physical and physiological issues [28].

The MQ4 score designates the weighting placed on tenderness, juiciness and flavour liking by consumers when they give a satisfaction score [18]. All age cohorts gave a higher (*p* < 0.05) MQ4 score to the Tender genotype compared to the Tough genotype. The MQ4 scores for the Tender genotype samples (Table 2) are similar to those found by Chong et al. (2019) [18], which ranged from 60.9 to 64.7 for 24-month old tenderstretch bulls, straight hung steers, and tenderstretch steers. The 65+ group also scored the Tender genotype samples higher than the Tough genotype samples, but not significantly, perhaps for the same reasons as outlined previously regarding attribute hedonics and loss of sensory acuity in texture perception. In Northern Ireland, the formula for MQ4 is 0.2 TE + 0.1 JU + 0.4 FL + 03 OL, with threshold scores set at 38, 60 and 77 [29]. Farmer at al. [29,30] found that Northern Irish consumers put more emphasis on flavour liking and had a lower standard for “satisfactory” and “better than everyday” beef compared to Australian consumers.

The association between animal genotype and the different sensory quality measures assessed by both trained panels is presented in Table 3. Numerically, the Tender genotype consistently scored superior to the Tough genotype, although the difference was only significant for Sensory Location 1, where the Tender genotype scored higher for tenderness, juiciness and flavour; the difference approached significant (*p* = 0.06) for chewiness in Sensory Location 2.

## 4. Implications

Previous studies have compared the phenotypic performance of cattle differing in genetic merit [31,32,33,34]; the hypothesis of the present study was that a detectable difference in meat tenderness should indeed exist between the two genotypes of cattle divergent in the genetic merit for tenderness. In fact, the observed mean difference in the tenderness score between both genotypes, when assessed by the trained panels (0.22 to 0.37), was larger than the expectation of 0.13 based on the difference in EBV of the heifers themselves (i.e., average of the EBV of the sire and dam). Added to the fact that the population of sires selected were not as divergent as they could be in regard to both within- and between breed variability, the actual difference in tenderness scores in the population as a whole could, in fact, be considerably more. This is hugely important given that the small genetic differences in tenderness among sires in the present study were actually clearly detected by consumers.

Although the reliability of the EBVs of individual animals for slaughter can be low, the reliability of the mean of a group can be high [12]. Hence, results from the present study clearly demonstrate the ability to stratify animals on genetic merit based solely on ancestral information in the pursuit of meat products differing in (detectable) tenderness. Supplementing the ancestry information with dense genomic data [35,36] will help augment the accuracy of the EBVs.

Another encouraging feature of the results from the present study was the favourable scoring for the other quality assessments of the meat samples other than just tenderness. Superior assessment for the other sensory qualities, however, is not unexpected given the moderate to strong genetic correlations between tenderness and both juiciness and flavour in cattle [13]. Hence, the alleles associated with greater tenderness are either those associated with improvements in other metrics or are co-inherited with those favourable alleles.

While the core objective of the present study was to validate genetic evaluations as a means of potentially stratifying products at the point of sale, an additional outcome is the proof that genetic selection for improved tenderness should result in the mean tenderness of a population shifting over time (assuming no change in management). Breeding has a particular advantage of being sustainable, rapid, cumulative and permanent. Several breeding goals exist in the dairy [37], beef [31,38] and beef-on-dairy industries [39]. These breeding goals are comprised of many traits thought to influence profit, thus providing a proven [32,38,40] vehicle to achieving genetic gain in a number of traits simultaneously, even if antagonistically correlated. Inclusion of meat sensory traits in such breeding goals is crucial to achieve generation-on-generation gains in these metrics.

## 5. Conclusions

The critical outcome from this study is that the estimated genetic merit of individuals, even animals at birth, can be used as a screening tool for tenderness and other meat quality traits, which, in turn, can be detected by consumers. Consumers significantly liked the Tender genotype samples for the liking of tenderness, liking of juiciness, liking of flavour and overall acceptability compared to the Tough genotype samples. Additionally, the Tender genotype, besides scoring better for tenderness, also scored better for juiciness, flavour and overall acceptance. All age cohorts (18–65+) scored the Tender genotype samples higher for liking of tenderness compared to the Tough genotype samples, as well as displaying this trend for the liking of juiciness, liking of flavour and overall acceptance in general across the consumer age spectrum (35–44, 65+ not significantly). MQ4 data were significantly higher for the Tender genotype samples compared to the Tough genotype samples for all age cohorts except the 65+ group. Overall, these data align with the trained panel data, particularly for Location 1, with significantly higher scores for tenderness, juiciness and flavour attributes for the Tender genotype samples.

## Figures and Tables

**Table 1:** Sensory hedonic mean data for each consumer age cohort with standard error of the mean (SEM).

			Liking of Tenderness					Liking of Aroma				
Age	No.		Tender Genotype		SEM	Tough Genotype	SEM	Tender Genotype	SEM	Tough Genotype		SEM
18–24	157		6.33 ^a^****		0.15	4.90 ^b^	0.19	6.28 ^NS^	0.27	5.84		0.15
25–34	146		6.98 ^a^*		0.25	5.93 ^b^	0.38	6.71 ^NS^	0.15	6.48		0.31
35–44	97		6.65 ^a^***		0.21	5.56 ^b^	0.25	6.07 ^NS^	0.21	5.87		0.22
45–54	63		6.47 ^a^***		0.24	5.37 ^b^	0.26	6.48 ^NS^	0.21	6.59		0.88
55–64	67		6.90 ^a^****		0.22	5.44 ^b^	0.28	5.86 ^NS^	0.23	5.41		0.21
65+	20		6.59 ^a^*		0.49	5.02 ^b^	0.49	5.35 ^NS^	0.37	4.92		0.44
	**Liking of** **Juiciness**				**Liking of** **Flavour**				**Overall** **Acceptability**			
**Age**	**Tender Genotype**	**SEM**	**Tough Genotype**	**SEM**	**Tender Genotype**	**SEM**	**Tough Genotype**	**SEM**	**Tender Genotype**	**SEM**	**Tough Genotype**	**Tough Genotype**
18–24	5.62 ^a^***	0.16	4.75 ^b^	0.18	6.20 ^a^****	0.14	5.34 ^b^	0.17	6.19 ^a^****	0.14	5.29 ^b^	0.16
25–34	5.87 ^a^***	0.17	5.00 ^b^	0.19	6.27 ^a^*	0.15	5.81 ^b^	0.15	6.37 ^a^****	0.14	5.57 ^b^	0.15
35–44	5.71 ^NS^	0.23	5.22	0.24	6.50 ^NS^	0.22	6.05	0.22	6.35 ^NS^	0.22	5.94	0.21
45–54	6.12 ^a^***	0.24	4.67 ^b^	0.27	6.44 ^a^***	0.20	5.60 ^b^	0.25	6.55 ^a^ ***	0.20	5.73 ^b^	0.24
55–64	5.69 ^a^*	0.27	4.91 ^b^	0.26	6.09 ^a^*	0.22	5.44 ^b^	0.25	6.19 ^a^*	0.22	5.56 ^b^	0.25
65+	4.66 ^NS^	0.57	4.10	0.51	5.44 ^NS^	0.43	5.47	0.31	5.68 ^NS^	0.47	5.31	0.41

No. = Number of consumer observations per genotype treatment. ^NS^ = Non-significant. Lower case letters ^(a, b)^ denote significant differences between columns. * = *p* < 0.05, ** = *p* < 0.01 *** = *p* < 0.001, **** = *p* < 0.0001.

**Table 2:** MQ4 scores for Tough and Tender genotypes across consumer demographics.

Age	Tender Genotype	SEM	Tough Genotype	SEM
18–24	61.77 ^a^****	1.26	51.36 ^b^	1.54
25–34	64.70 ^a^***	1.48	56.91 ^b^	1.84
35–44	64.22 ^a^**	1.97	57.85 ^b^	2.09
45–54	64.50 ^a^**	2.00	54.79 ^b^	2.31
55–64	63.24 ^a^**	2.01	54.24 ^b^	2.29
65+	57.78 ^NS^	4.29	51.49 ^NS^	3.61

^NS^ = Non-significant. Lower case letters ^(a, b)^ denote significant differences between rows. Mean scores and SEM. * = *p* < 0.05, ** = *p* < 0.01 *** = *p* < 0.001, **** = *p* <0.0001.

**Table 3:** Trained sensory panel data: least squares means and standard error of the difference (SED) for the different sensory attributes measured in Location 1 (scale 1 (poor) to 9 (good)) and Location 2 (scale 1 (poor) to 10 (good)) for both the genetically Tender and Tough genotypes.

Trait	Tender	Tough	SED	*p*-Value
Location 1				
Tenderness	7.00	6.63	0.184	≤0.05
Juicy	6.91	6.34	0.180	≤0.01
Flavour	7.04	6.75	0.124	≤0.05
Location 2				
Tenderness	6.95	6.73	0.158	0.18
Juicy	6.36	6.34	0.168	0.93
Flavour	6.77	6.73	0.159	0.78
Chewy	2.91	3.25	0.173	0.06

## Data Availability

Not applicable.
